# Neutralization of SARS-CoV-2 Omicron and Delta Variants in Relation to Vaccine-Induced Antibody Levels in Kidney Transplant Recipients and Healthy Controls

**DOI:** 10.1128/spectrum.01314-22

**Published:** 2022-09-28

**Authors:** Rune M. Pedersen, Line L. Bang, Ditte S. Tornby, Helene Kierkegaard, Anna C. Nilsson, Isik S. Johansen, Thomas V. Sydenham, Thøger G. Jensen, Ulrik S. Justesen, the COVAC-TX Study Group, Claus Bistrup, Thomas E. Andersen

**Affiliations:** a Department of Clinical Microbiology, Odense University Hospital and Research Unit for Clinical Microbiology, University of Southern Denmark, Odense, Denmark; b Department of Biochemistry and Immunology, Lillebaelt Hospital, University Hospital of Southern Denmark, Kolding, Denmark; c Department of Clinical Immunology, Odense University Hospital and Research Unit for Clinical Immunology, University of Southern Denmark, Odense, Denmark; d Department of Infectious Diseases, Odense University Hospital and Research Unit for Infectious Diseases, University of Southern Denmark, Odense, Denmark; e Department of Nephrology, Odense University Hospital and the Nephrology Research Unit, University of Southern Denmark, Odense, Denmark; National Institutes of Health

**Keywords:** COVID-19, Delta, kidney transplant recipients, Omicron, PRNT, SARS-CoV-2, neutralizing antibodies, vaccines

## LETTER

High levels of anti-SARS-CoV-2 antibodies are, in addition to a robust T cell mediated immunity, required to provide adequate protection against COVID-19. The level of antibodies (Ab) raised in response to the COVID-19 vaccines is a useful indicator of the acquired SARS-CoV-2 neutralizing capacity and hence risk of severe COVID-19 especially in immune suppressed patients such as kidney transplant recipients (KTRs) (1). Since the roll-out of the vaccines, however, SARS-CoV-2 variants have emerged which partly evade vaccine-induced immunity. In the last half of 2021, the Delta variant was dominating and in recent months the Omicron has outcompeted Delta in many countries (2). The pronounced immune-evasion by these variants greatly influences the neutralization-equivalent thresholds in the available commercial immunoassays, rendering current recommended limits unreliable as they refer to early SARS-CoV-2 isolates from 2020 close to the Wuhan-Hu-1 reference strain (3). To update these limits and provide a reference for neutralization of current variants, we conducted live virus neutralization assays with authentic SARS-CoV-2 Delta and Omicron isolates and compared these results with the Ab levels measured on 3 widely used commercial immunoassay platforms.

As the gold standard for assessing humoral protection against SARS-CoV-2, we used 90% plaque reduction neutralization tests (PRNT_90_) as previously described (4). PRNT_90_ were performed with clinical Delta and Omicron isolates (genomic details: see Supplemental Material) and using blood plasma from 57 KTRs and 20 age matched healthy controls collected 4 weeks after the second BNT162b2 vaccination (4). Plasma samples were analyzed for spike Ab on the immunoassay platforms Euroimmun (EUROIMMUN), Liaison (DiaSorin), and Vitros Quantitative (Ortho), according to manufacturer’s recommendations (Table 1). All included persons were nucleocapsid antibody negative indicating that none had previously been infected (data not shown).

**TABLE 1:** Sensitivity and specificity for manufacturer-recommended and new suggested thresholds of antibody levels as measured on 3 commercial SARS-CoV-2 spike antibody immunoassay platforms^*a*^

SARS-CoV-2 strain		Threshold		Threshold x5		Threshold x100	
and immunoassay	No.	Sensitivity	Specificity	Sensitivity	Specificity	Sensitivity	Specificity
Ancestral strain (B.1.118)							
Euroimmun^*b*^ (95% CI^*c*^)	74	**100%** **(91–100%)**	**95%** **(82–99%)**	84% (68–94%)	100% (91–100%)	8% (17–22%)	100% (91–100%)
Delta strain (B.1.617.2)							
Vitros quan^*d*^ (95% CI^*c*^)	76	100% (91–100%)	80% (69–92%)	**97%** **(83–100%)**	**96%** **(85–99%)**	30% (15–49%)	100% (92–100%)
Liaison^*e*^ (95% CI^*c*^)	76	100% (91–100%)	80% (69–92%)	**97%** **(83–100%)**	**98%** **(88–100%)**	N/A^*f*^	N/A^*f*^
Euroimmun^*b*^ (95% CI^*c*^)	75	100% (89–100%)	80% (65–90%)	**97%** **(83–100%)**	**98%** **(88–100%)**	10% (2–26%)	100% (92–100%)
Omicron strain (BA.1)							
Vitros quan^*d*^ (95% CI^*c*^)	50	100% (29–100%)	32% (19–47%)	100% (29–100%)	47% (32–62%)	**100%** **(29–100%)**	**89%** **(77–96%)**
Liaison^*e*^ (95% CI^*c*^)	50	100% (29–100%)	28% (16–43%)	100% (29–100%)	49% (34–64%)	**N/A** ^*f*^	**N/A** ^*f*^
Euroimmun^*b*^ (95% CI^*c*^)	51	100% (40–100%)	28% (16–43%)	100% (40–100%)	47% (32–62%)	**50%** **(7–93%)**	**96%** **(85–99%)**

*^a^*The threshold limits provided by the manufacturers are: Vitros quan, 17.8 binding antibody units/mL; Liaison, 34.8 binding arbitrary units/mL; Euroimmun, 24 binding antibody units/mL. Vitros quan, Liaison and Euroimmun are all FDA approved assays. Suggested more optimal threshold limits for the individual strains are marked in bold. The numbers of analyzed plasma samples are explained in Supplemental Material Fig. S1.

*^b^*Euroimmun, EUROIMMUN Anti-SARS-CoV-2 QuantiVac ELISA (IgG).

*^c^*CI, confidence interval.

*^d^*Vitros quan, the CD VITROS quantitative Anti–SARS-CoV-2 IgG immunoassay.

*^e^*Liaison, the Liaison SARS-CoV-2 TrimericS IgG assay.

*^f^*N/A, not applicable (above detection threshold).

**FIG 1:**
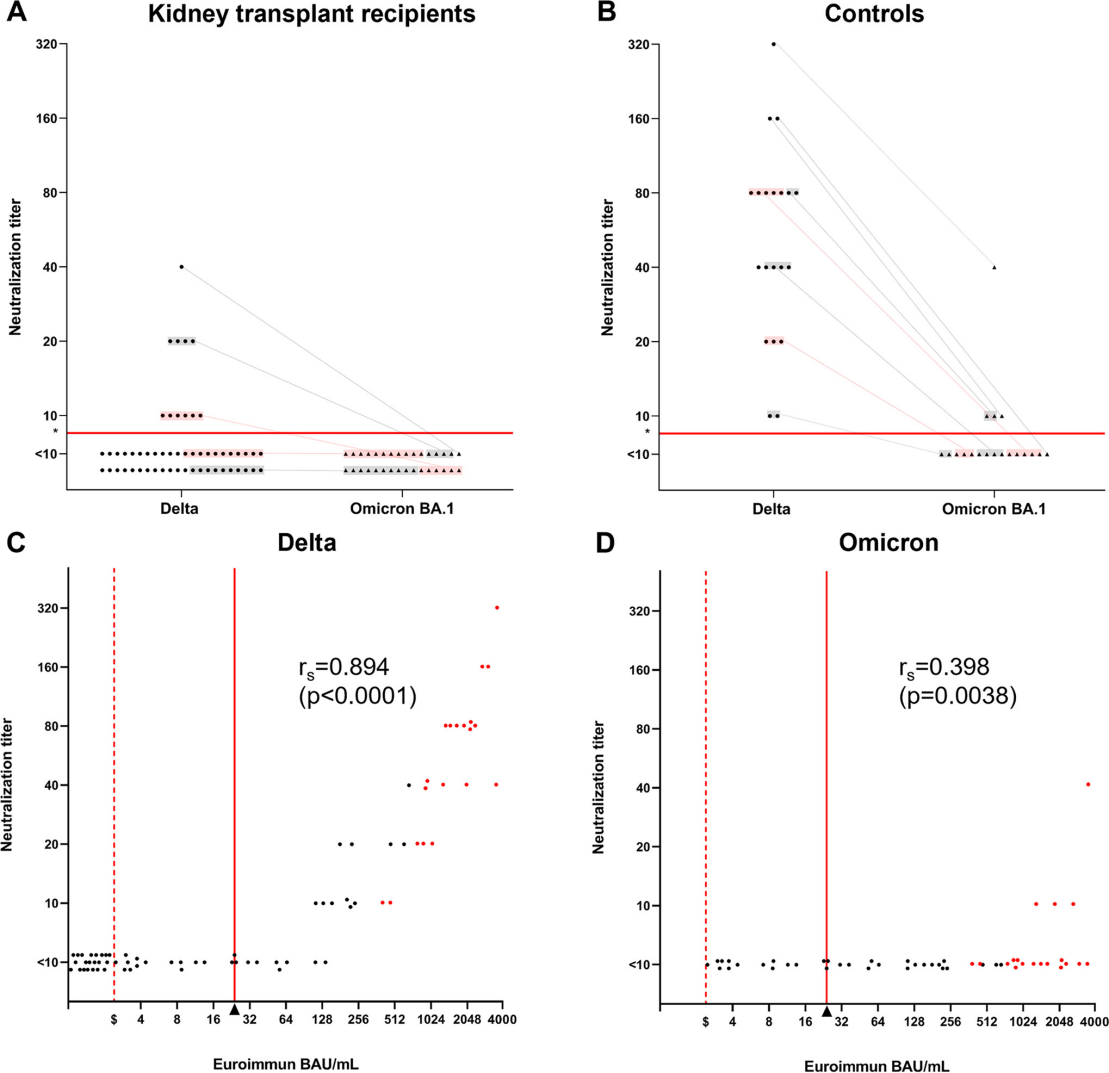
Neutralization of SARS-CoV-2 Delta and the Omicron and the corresponding antibody levels among vaccinated kidney transplant recipients and age matched controls. (A) Neutralization titers (PRNT_90_) of KTRs against the Delta strain (*n* = 57) and the Omicron strain (sublineage BA.1) (*n* = 32) 4 weeks after the second dose of BNT162b2. The same plasma samples are connected by gray lines. (B) Neutralization titers of healthy controls against the Delta strain (*n* = 20) and the Omicron strain (*n* = 19) 4 weeks after the second dose of BNT162b2. (C) Dot plot demonstrating the performance of the EUROIMMUN Anti-SARS-CoV-2 QuantiVac ELISA (IgG) (EUROIMMUN) for predicting the neutralization titer against the Delta strain (*n* = 77). Samples from KTRs are shown as black dots, whereas samples from controls are shown as red dots. The detected levels correlated with the Delta strain neutralization titers, ρ = 0.894 (*P* < 0.0001, Spearman’s correlation) (D) Dot plot demonstrating the performance of EUROIMMUN for predicting the neutralization titer against the Omicron strain (*n* = 51). Samples from KTRs are shown as black dots, whereas samples from controls are showed as red dots. The detected levels correlated with the Omicron strain neutralization titers, r_s_ = 0.398 (*P* = 0.0038, Spearman's correlation). *Neutralization threshold limit indicated with a red horizontal line in (A) and (B). ^Δ^Cut off of 24 binding antibody units/mL provided by the manufacturer indicated with red vertical lines in (C) and (D). ^$^Lower level of detection indicated with a dotted red line in (C) and (D). PRNT_90_, 90% plaque reduction neutralization test; Ab, antibody; KTRs, kidney transplant recipients; SARS-CoV-2, severe acute respiratory syndrome coronavirus 2.

The sensitivity and specificity of the Ab cutoff levels as measured on the commercial assays in relation to the neutralization thresholds as measured by PRNT_90_ is shown in Table 1. The neutralizing capacities of blood plasma against the Omicron and Delta variants are shown in Fig. 1A and B. Ab levels and neutralizing capacities of this cohort against the ancestral strain (B.1.118) are available in a previous report (4). The Ab levels detected in the Euroimmun assay correlated with the Delta strain neutralization titers, ρ = 0.894 (*P* < 0.0001, Spearman's correlation [Fig. 1C]), and the Omicron strain neutralization titers, ρ = 0.398 (*P* = 0.0038, Spearman's correlation [Fig. 1D]). As shown in Table 1, neutralization titers against Delta can be anticipated by raising the predefined threshold levels 5-fold, whereas raising the threshold level approximately 100-fold better reflects neutralization of Omicron. It should be noted, however, that due to the low number of Omicron neutralizing individuals this threshold is associated with uncertainty, as also indicated by the low sensitivity for Euroimmun when using this threshold value (Table 1). The data furthermore shows that while Ab levels in 20/55 (36%) of the KTRs are above the manufacturer-recommended threshold indicating neutralizing capacity (Euroimmun), of these, only 11/20 (55%) and 0/20 (0%) neutralize Delta and Omicron, respectively.

The current study is based on blood samples from two-times BNT162b2 vaccinated individuals. Currently, booster doses have been rolled out in many countries which leads to at least transient increase in Ab levels and corresponding increase in vaccine effectiveness (5). Assuming equal affinity of the Abs after booster doses, the translation of Ab levels to neutralization of the variants provided here should remain valid. Sublineage BA.2 of Omicron now replaces the original Omicron BA.1 (B.1.1.529) lineage, however, according to own results, BA.2 and BA.1 are neutralized equally in the same serum samples, suggesting that the limits indicated for BA.1 in Table 1 is valid for BA.2 as well (6). The presented data is mainly relevant for assessment of the humoral protection against SARS-CoV-2 variants in immune suppressed patients such as KTRs, where the effect of the vaccines can be questioned. Moreover, it is relevant to point out that patients that have recovered from infection, especially with newer variants, will likely neutralize these variants more effectively and hence have lower neutralization thresholds than indicated for the vaccinated-only cohort analyzed here. As additional vaccine boosters, according to current recommendations, are given to immune suppressed patients, Ab levels will be expected to increase, for some reaching levels that should be neutralizing also against newer SARS-CoV-2 variants.
