# Supplementary material for: Neutralization of SARS-CoV-2 Omicron and Delta Variants in Relation to Vaccine-Induced Antibody Levels in Kidney Transplant Recipients and Healthy Controls
Source: Microbiol Spectr. 2022 Sep 28;10(5):e01314-22. doi: 10.1128/spectrum.01314-22 (PMC9603620; doi:10.1128/spectrum.01314-22)
Supplement: Supplemental file 1 — Table S1, Figure S1. Download spectrum.01314-22-s0001.pdf, PDF file, 0.3 MB [file spectrum.01314-22-s0001.pdf]

Supplemental file S1

Supplemental Methods

RNA extraction was performed using the Chemagic Viral DNA/RNA 300 Kit H96 kit on the Chemagic 360 instrument (PerkinElmer, Skovlunde, Denmark) with 300 µl aliquot swab material. Genome sequencing followed the ARTIC network nCoV-2019 sequencing protocol v3 (LoCost) using V4 primers, with runs of 23-47 samples and one - three negative control on a MinION sequencing instrument (Oxford Nanopore Technologies, Oxford, UK) with MinKNOW v 21.02.1 and live basecalling with Guppy v 4.3.4 [S1]. A consensus genome sequence was generated by following the ARTIC-network ncov bioinformatics protocol by aligning reads to the Wuhan-Hu-1 reference (Genbank: MN908947). Lineage and mutation calls were performed using Pangolin v 3.1.17 with pangoLEARN database v06-12-2021 and Nextclade v. 0.14.4 [S2,S3]. Sequences for the Omicron, Delta and the ancestral strain are available at GenBank, accession no. ON055874, ON055856, and ON055855, respectively.

**Strain**	**Ancestral strain**	**Delta strain**	**Omicron strain**
Lineage	B.1.118	B.1.617.2	BA.1
Missing nucleotides	550	383	536
AA mutations			
ORF1a	ORF1a:T265I	**ORF1a:P1640L**	ORF1a:K856R
		**ORF1a:K141-**	ORF1a:S2083I
		**ORF1a:S142-**	ORF1a:L2084-
		**ORF1a:F143-**	ORF1a:A2710T
		**ORF1a:G2987S**	ORF1a:T3255I
		**ORF1a:A3209V**	ORF1a:P3395H
		**ORF1a:V3718A**	**ORF1a:L3674F**
			ORF1a:S3675-
			ORF1a:G3676-
			ORF1a:F3677-
			ORF1a:I3758V
		**ORF1a:T3750I**	
ORF1b	ORF1b:P314L	**ORF1b:P314L**	ORF1b:P314L
	ORF1b:T792N	**ORF1b:G662S**	ORF1b:I1566V
		**ORF1b:P1000L**	
S	**S:N74K**	S:T19R	S:A67V
	S:D614G	**S:G75V**	S:H69-
		S:G142D	S:V70-
		**S:E156G**	S:T95I
		**S:F157-**	S:G142D
		**S:R158-**	S:V143-

		**S:A222V**	S:Y144-
		S:L452R	S:Y145-
		S:T478K	S:N211I
		**S:D614G**	S:L212-
		S:P681R	S:G339D
		S:D950N	S:S371L
			S:S373P
			S:S375F
			S:S477N
			S:T478K
			S:E484A
			S:Q493R
			S:G496S
			S:Q498R
			S:N501Y
			S:Y505H
			S:T547K
			S:D614G
			S:H655Y
			S:N679K
			S:P681H
			**S:N764K**
			S:D796Y
			S:N856K
			S:Q954H
			S:N969K
			S:L981F
ORF3a	**ORF3a:P36L**	ORF3a:S26L	
	ORF3a:Q57H	**ORF3a:W45L**	
E			E:T9I
M		M:I82T	M:D3G
			M:Q19E
			M:A63T
ORF7a	**ORF7a:F6X**	ORF7a:V82A	
		ORF7a:T120I	
ORF8		**ORF8:D119-**	
		**ORF8:F120-**	
N	N:T417I	**N:M1X**	N:P13L
		N:D63G	N:E31-
		N:R203M	N:R32-
		N:D377Y	N:S33-
			N:R203K
			N:G204R
ORF9b		**ORF9b:N28S**	**ORF9b:N28-**

		**ORF9b:T60A**	**ORF9b:A29-**
			**ORF9b:V30-**
			**ORF9b:P10S**
			**ORF9b:E27V**

Supplementary Table 1: Genomic analysis of SARS-CoV-2 strains. Identified mutations in the Delta and Omicron SARS-CoV-2 strains used in PRNT assays, and the reference ancestral strain used in Table 1. Non-strain defining mutations are highlighted in bold.

Estimation of optimal thresholds

The sensitivity and specificity were calculated for the commercial platforms using the PRNT90 titers as the reference. Here, sensitivity was estimated with the following equation: number of true positives/(number of true positives + number of false negatives). Specificity was estimated with the following equation: number of true negatives/(number of true negatives + number of false positives). The suggested more optimal Ab cut off levels corresponding to neutralization of the Delta and the Omicron variant of SARS-CoV-2 were initially estimated based on visual inspection of the Ab values as a function of the neutralization titers in a dot plot. Subsequently, the Ab level yielding the maximum sum of sensitivity plus specificity was chosen as the more optimal level.

PRNT of two additional Delta strains

To assess whether the non-strain defining mutations in the Delta strain (see Supplemental Table 1) gave rise to unusual PRNT levels, two additional Delta strain isolates were analyzed in 6 patient plasma samples which showed the same PRNT result (data not shown). These Delta isolates contained the following genomic changes:

AY.42:
M:I82T,N:D63G,N:R203M,N:G215C,N:D377Y,ORF1a:V665I,ORF1a:A1306S,ORF1a:P2046L,ORF1a:P2287S,ORF1a:V2930L,ORF1a:T3255I,ORF1a:T3646A,ORF1b:P314L,ORF1b:G662S,ORF1b:D815Y,ORF1b:P1000L,ORF1b:E1288D,ORF1b:A1918V,ORF3a:S26L,ORF3a:G224R,ORF7a:V82A,ORF7a:T120I,ORF7b:T40I,ORF9b:T60A,S:T19R,S:R158G,S:L452R,S:T478K,S:D614G,S:P681R,S:T719I,S:D950N,S:R1014T;ORF8:D119-,ORF8:F120-,S:E156-,S:F157-

B.1.617.2:
M:I82T,N:D63G,N:T135I,N:R203M,N:G215C,N:D377Y,ORF1a:H1141Y,ORF1a:A1306S,ORF1a:P2046L,ORF1a:V2930L,ORF1a:T3255I,ORF1a:T3646A,ORF1b:P314L,ORF1b:G662S,ORF1b:P1000L,ORF1b:A1918V,ORF3a:S26L,ORF7a:V82A,ORF7a:T120I,ORF7b:T40I,ORF9b:A57V,ORF9b:T60A,S:T19R,S:T95I,S:G142D,S:R158G,S:L452R,S:T478K,S:D614G,S:P681R,S:D950N,S:E1202Q;ORF8:D119-,ORF8:F120-,S:E156-,S:F157-

Plasma sample flow chart

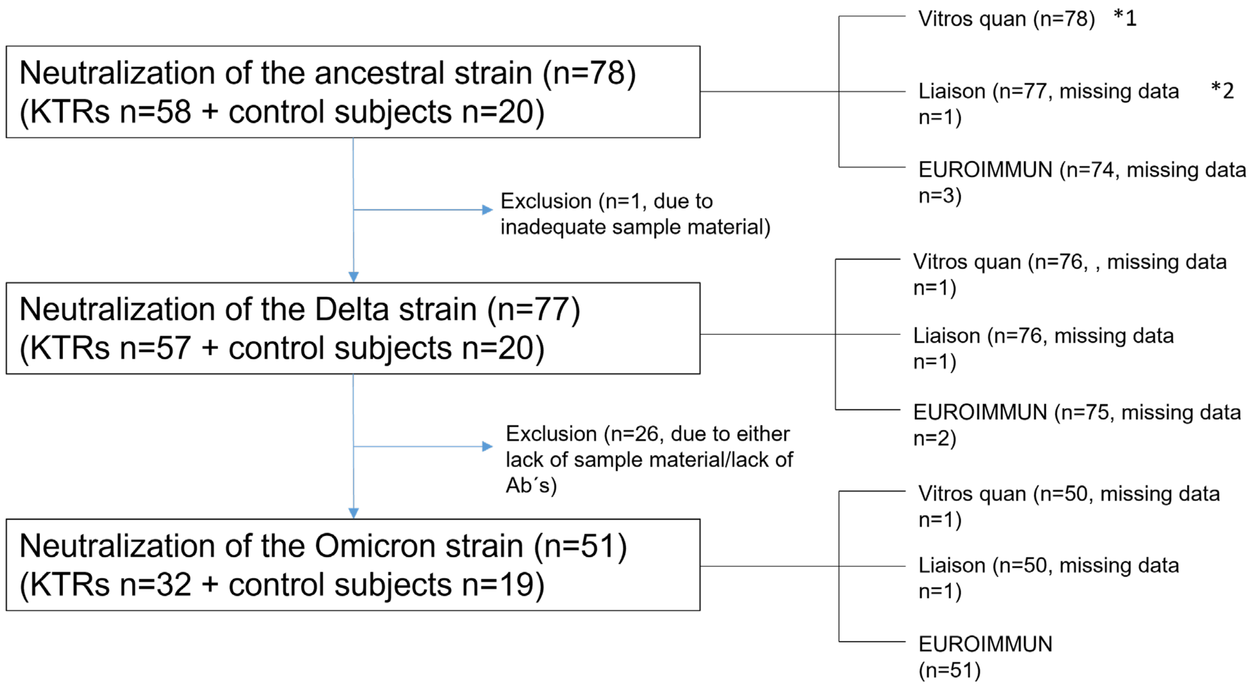

Supplementary Figure 1

Flow chart indicating the number of collected and analysed patient plasma samples.

*1 and *2: Data available in [S4].

S1. Tyson JR, James P, Stoddart D, Sparks N, Wickenhagen A, Hall G, et al. Improvements to the ARTIC multiplex PCR method for SARS-CoV-2 genome sequencing using nanopore. bioRxiv. 2020 Sep 4;2020.09.04.283077.

S2. O'Toole Á, Scher E, Underwood A, Jackson B, Hill V, McCrone JT, et al. Assignment of epidemiological lineages in an emerging pandemic using the pangolin tool. Virus Evol [Internet]. 2021 Oct 1 [cited 2021 Oct 7];7(2). Available from: https://doi.org/10.1093/ve/veab064

S3. Hadfield J, Megill C, Bell SM, Huddleston J, Potter B, Callender C, et al. Nextstrain: real-time tracking of pathogen evolution. Bioinformatics. 2018 Dec 1;34(23):4121–3.

S4. Pedersen RM, Bang LL, Tornby DS, et al. The SARS-CoV-2-neutralizing capacity of kidney transplant recipients 4 weeks after receiving a second dose of the BNT162b2 vaccine. Kidney Int. 2021;100:1129–1131.
